# Method to Eliminate Flux Linkage DC Component in Load Transformer for Static Transfer Switch

**DOI:** 10.1155/2014/724529

**Published:** 2014-07-17

**Authors:** Yu He, Chengxiong Mao, Jiming Lu, Dan Wang, Bing Tian

**Affiliations:** State Key Laboratory of Advanced Electromagnetic and Technology, Huazhong University of Science and Technology, Wuhan 430074, China

## Abstract

Many industrial and commercial sensitive loads are subject to the voltage sags and interruptions. The static transfer switch (STS) based on the thyristors is applied to improve the power quality and reliability. However, the transfer will result in severe inrush current in the load transformer, because of the DC component in the magnetic flux generated in the transfer process. The inrush current which is always 2~30 p.u. can cause the disoperation of relay protective devices and bring potential damage to the transformer. The way to eliminate the DC component is to transfer the related phases when the residual flux linkage of the load transformer and the prospective flux linkage of the alternate source are equal. This paper analyzes how the flux linkage of each winding in the load transformer changes in the transfer process. Based on the residual flux linkage when the preferred source is completely disconnected, the method to calculate the proper time point to close each phase of the alternate source is developed. Simulation and laboratory experiments results are presented to show the effectiveness of the transfer method.

## 1. Introduction

DUAL power sources are available in many companies and industry parks to improve the power supply reliability. And the static transfer switch (STS) [1–9] is applied to achieve the fast transfer from the preferred source to the alternate source.

In industrial application, there is always a transformer on the load side of STS [7, 10, 11]. The DC component in flux linkage of the load transformer will be generated during the transfer interval. The transfer achieved by the STS will result in severe inrush current in the load transformer. The inrush current which is always 2–30 p.u. can cause the disoperation of protective relay and influence the power quality [10].

The method to reduce inrush current in the energizing process of the transformer is discussed in many articles [12–18]. The sequential switching scheme is proposed to reduce the inrush current when the transformer is energized [12–14].

The paper [7] proposes a way to reduce the inrush current in the STS system. By transferring the related phases when the estimated peak flux linkage of the load transformer is under the predetermined level, the flux linkage DC component can be limited. Since the transfer scheme cannot eliminate the DC component of the flux linkage theoretically, there is still significant DC component contained in the flux linkage after the transfer. And the transient process of the flux linkage of each winding in the transfer process may not be fully analyzed.

This paper analyzes the transient process of the flux linkage in every stage during the transfer interval. The transient process of the flux linkage of each winding in the load transformer during the transfer interval is analyzed.

The method to calculate the proper time point to transfer each phase based on the transient process of the flux linkage is developed. In addition, this paper discusses whether there is best time instant to transfer the third phase. In order to test that the transfer method is functional, the results of the simulation and the laboratory experiments are presented.

## 2. Operation Principles

**Figure 1:**
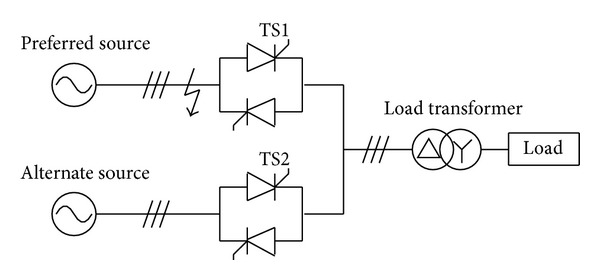
STS and the load transformer.

The one-line diagram of STS and the load transformer is presented in Figure 1. TS1 and TS2 are antiparallel thyristor switches connected to preferred source and alternate source, respectively. And on the load side there is a transformer with the three-phase load.

### 2.1. Stages during the Transfer Process

In order to analyze the transient process of the flux linkage of the load transformer during the transfer interval, it is necessary to explain the stages during the transfer process.

In the transfer process, the paralleling of the two sources is forbidden. The “break before make” transfer strategy is adopted. The alternate source can only connect to the load when the preferred source is completely disconnected.

**Figure 2:**
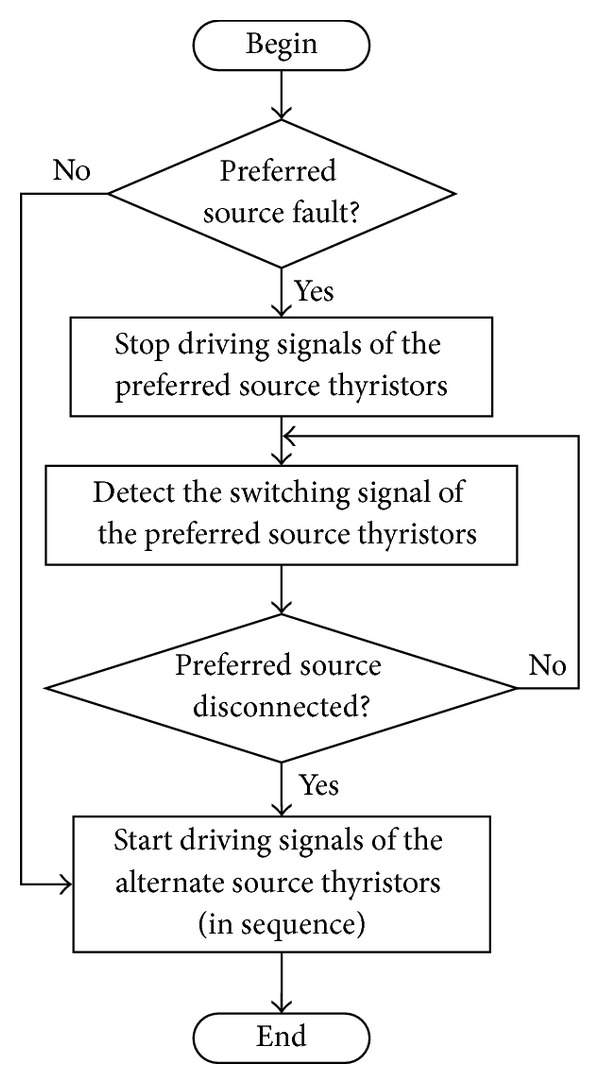
Control flowchart of the transfer process.

The control flowchart of the “break before make” transfer strategy is shown in Figure 2. The transfer process consists of four stages below.

*(1) Fault Detection Stage*. After the preferred source faults, STS needs some time to detect the fault. When the fault of the preferred source is detected, STS stops the driving signals of the preferred source thyristors.

*(2) Preferred Source Disconnecting Stage*. After the driving signals of the preferred source thyristors are stopped, the current will be blocked at the first current zero crossing point. Three phases of the preferred source will be disconnected at different time. 

*(3) Off Stage*. In order to avoid the paralleling of two sources in the transfer process, STS needs to detect the switching signal of the preferred source thyristors before starting the driving signal of the alternate source thyristors. So there will be a time interval that the load connects to neither of the sources.

*(4) Alternate Source Transferring Stage*. When the STS detects that the preferred source is completely disconnected, the driving signals of alternate source thyristors start. But if the three phases of the alternate source transfer at the same time, there is significant flux linkage DC component contained in the load transformer after the transfer which will result in severe inrush current. The transfer of the alternate source should be operated in sequence according to the flux linkage to eliminate the DC component of the flux linkage.

### 2.2. Generation of the Flux Linkage DC Component

The DC component of the flux linkage after the transfer is the reason why the inrush current emerges. The generation of the DC component in the transfer process is discussed in this part.

**Figure 3:**
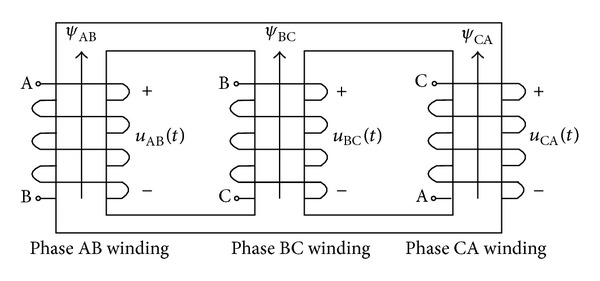
Connection of the primary windings of the delta/wye transformer.

The analysis is based on the delta/wye transformer which is widely used in the distribution grid. The connection of the primary windings in the delta/wye transformer is shown in Figure 3. The magnetic flux linkage of the phase AB winding can be expressed as below:

(1) $$\begin{array}{c} \psi_{\mathrm{tr},\text{AB}}\left(t\right)=\int u_{\text{AB}}\left(t\right)dt. \end{array}$$

The *u*(*t*) is the corresponding line voltage of the primary side of the transformer. Since the three windings of the primary side make up a circle, the voltages of the three windings follow the equation below:

(2) $$\begin{array}{c} u_{\text{AB},\mathrm{tr}}\left(t\right)+u_{\text{BC},\mathrm{tr}}\left(t\right)+u_{\text{CA},\mathrm{tr}}\left(t\right)=0. \end{array}$$

The flux linkages of the transformer satisfy the condition in

(3) $$\begin{array}{c} \psi_{\text{AB},\mathrm{tr}}\left(t\right)+\psi_{\text{BC},\mathrm{tr}}\left(t\right)+\psi_{\text{CA},\mathrm{tr}}\left(t\right)=0. \end{array}$$

**Figure 4:**
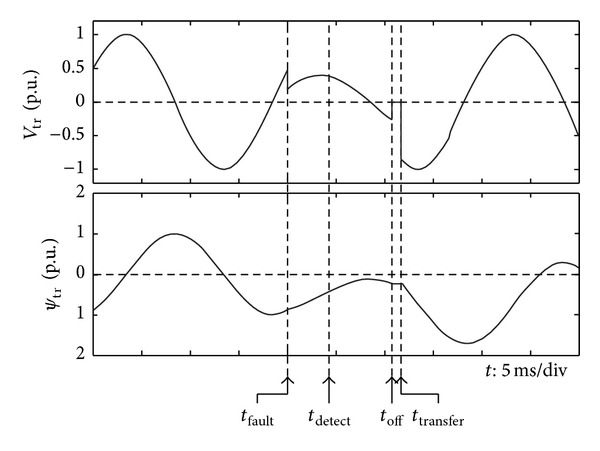
Transformer voltage and flux linkage during the transfer process.

Figure 4 illustrates the transient process of the line voltages of transformer and the flux linkage of the winding during the transfer interval.

The flux linkage after the transfer can be calculated by

(4) $$\begin{array}{c} \psi_{\mathrm{tr}}\left(t\right)={\psi_{\mathrm{tr}}|}_{t=t_{\text{fault}}}+\overset{t_{\text{transfer}}}{\underset{t_{\text{fault}}}{\int }}U_{\mathrm{tr}}\left(t\right)dt+\overset{t}{\underset{t_{\text{transfer}}}{\int }}U_{\text{alt}}\left(t\right)dt. \end{array}$$

During the off stage (*t*_*off*⁡_ < *t* < *t*_transfer_), the line voltages on the primary side of the transformer become zero. In this stage, the flux linkage in the load transformer remains constant.

(5) $$\begin{array}{c} \overset{t_{\text{transfer}}}{\underset{t_{\mathrm{off}}}{\int }}U_{\mathrm{tr}}\left(t\right)dt=0. \end{array}$$

This equation can be rewritten as below:

(6) ψtr⁡(t)=ψtr⁡|t=tfault+∫tfaulttoff⁡utr⁡(t)dt+∫ttransfertualt(t)dt=ψtr⁡|t=tfault+∫tfaulttoff⁡utr⁡(t)dt−ψalt|t=ttransfer+ψalt(t).

The flux linkage after the transfer can be expressed as the combination of the periodic component and the DC component in

(7) $$\begin{array}{c} \psi_{\mathrm{tr}}\left(t\right)=\psi_{\text{alt}}\left(t\right)+K. \end{array}$$

The DC component *K* can be expressed as below:

(8) $$\begin{array}{c} K={\psi_{\mathrm{tr}}\left(t\right)|}_{t=t_{\text{fault}}}+\overset{t_{\mathrm{off}}}{\underset{t_{\text{fault}}}{\int }}u_{\mathrm{tr}}\left(t\right)dt-{\psi_{\text{alt}}\left(t\right)|}_{t=t_{\text{transfer}}}. \end{array}$$

The *K* is the difference between two parts. The first part *ψ*_tr⁡_(*t*)|_*t*=*t*_fault__ + ∫_*t*_fault__^*t*_*off*⁡_^ *u*_tr⁡_(*t*)*dt* is the residual flux linkage after the preferred source is completely disconnected. The second part *ψ*_alt_(*t*)|_*t*=*t*_transfer__ is the prospective flux linkage of the alternate source at the transferring time instant.

The DC component of the flux linkage after the transfer is influenced by the fault detection time, the fault characteristics, the difference between two sources, the blocking time of the preferred source, and the transferring time instant of the alternate source.

### 2.3. The Transfer Time Instant of the First Two Phases

Based on the analysis in Section 2.2, in order to eliminate the DC component in the transfer process, we can only transfer the related phases when the residual flux linkage of the winding and the corresponding prospective flux linkage are equal.

**Figure 5:**
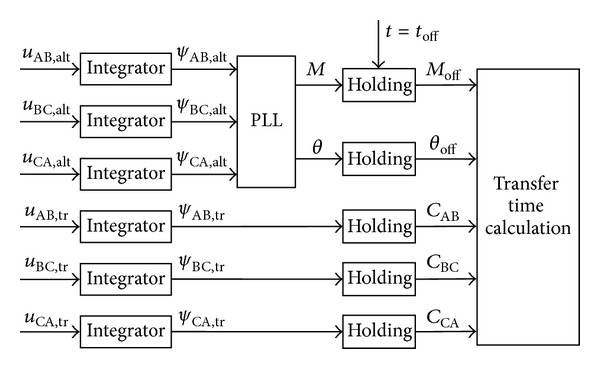
The block diagram of the flux linkage measuring.

The flux linkage measuring process is shown in Figure 5. The real-time residual flux linkages of three windings are acquired by integrating of the line voltages of the primary side of the transformer. When the off signal of the preferred source is detected, the integration results of the line voltages are the residual flux linkages of each winding. *C*_AB_, *C*_BC_, and *C*_CA_ are the residual flux linkages of the phase AB, BC, and CA winding of the load transformer when the preferred source is disconnected.

The prospective flux linkage of the alternate source is also acquired by integrating of the line voltage of the alternate source. The peak value and the phase angle of the prospective flux linkage of the alternate source can be calculated from the value of the line voltages of the alternate source. So the prospective flux linkage of the alternate source when the preferred source is completely disconnected can be shown as below:

(9) $$\begin{array}{c} \psi_{\text{alt}}\left(t\right)=M_{\mathrm{off}}\sin\left(\omega t+\theta_{\mathrm{off}}\right). \end{array}$$

*M*_*off*⁡_ and *θ*_*off*⁡_ are the peak value and the phase angle of the prospective flux linkage of the alternate source when the preferred source is completely disconnected.

The time instants to transfer each phase can be calculated by solving the equations below:

(10) $$\begin{array}{c} M_{\mathrm{off}}\sin\left(\omega t_{\text{AB}}+\theta_{\mathrm{off}}\right)=C_{\text{AB}},\\ M_{\mathrm{off}}\sin\left(\omega t_{\text{BC}}+\theta_{\mathrm{off}}-\frac{2\pi }{3}\right)=C_{\text{BC}},\\ M_{\mathrm{off}}\sin\left(\omega t_{\text{CA}}+\theta_{\mathrm{off}}+\frac{2\pi }{3}\right)=C_{\text{CA}}. \end{array}$$

In order to minimize the time of the off stage, the minimum value of the transfer time is chosen among the solutions of the three equations. And the related phases will transfer at that time instant.

**Figure 6:**
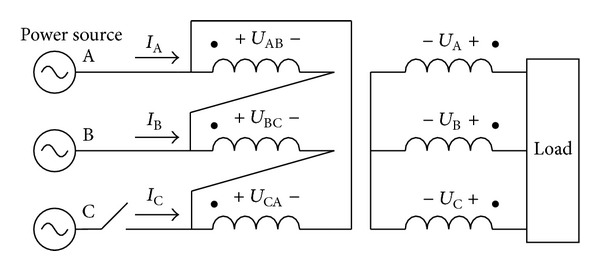
The two-phase-powered model of the delta/wye transformer.

After the first two phases transferred, the load transformer is powered by only two phases. The two-phase-powered model is shown in Figure 6.

Assume phase A and phase B of the alternate source are connected to the load transformer and phase C is disconnected. The voltage of the phase AB winding is the line voltage of the alternate source:

(11) $$\begin{array}{c} U_{\text{AB},\mathrm{tr}}=U_{\text{AB},\text{alt}}. \end{array}$$

And the phase BC winding and the phase CA winding are in series connections. Assume the parameters of each phase are the same. The voltages of the phase BC winding and the phase CA winding are equal:

(12) $$\begin{array}{c} U_{\text{CA},\mathrm{tr}}=U_{\text{BC},\mathrm{tr}}. \end{array}$$

Combining the condition in (2), the transformer voltages satisfy the condition in

(13) $$\begin{array}{c} U_{\text{AB},\mathrm{tr}}=-U_{\text{CA},\mathrm{tr}}-U_{\text{BC},\mathrm{tr}}. \end{array}$$

Then there will be the expression below:

(14) $$\begin{array}{c} U_{\text{BC},\mathrm{tr}}=U_{\text{CA},\mathrm{tr}}=-\frac{1}{2}U_{\text{AB},\mathrm{tr}}. \end{array}$$

**Figure 7:**
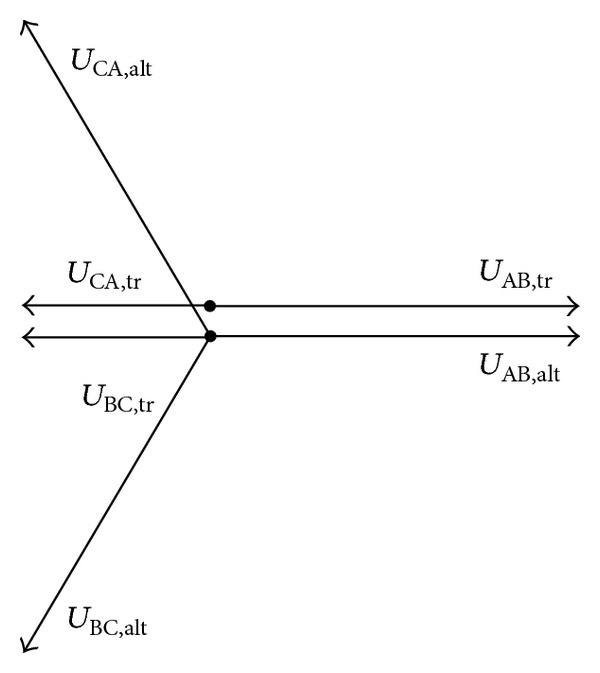
Vector diagram of the three-phase transformer voltages when two phases are connected.

The vector diagram of the transformer voltages when phase A and phase B are connected to the transformer is shown in Figure 7.

And the currents of each phase follow the equations below.

(15) $$\begin{array}{c} I_{\text{A}}=-I_{\text{B}},\\ I_{\text{C}}=0. \end{array}$$

### 2.4. The Transfer Time Instant of the Third Phase

If the phase A and phase B transfer first at the time instant that satisfies the condition in

(16) $$\begin{array}{c} \psi_{\text{AB},\text{alt}}\left(t\right)|{\;}_{t_{\text{transfer}}}=C_{\text{AB}}, \end{array}$$

the flux linkage of the phase AB winding can be expressed in

(17) $$\begin{array}{c} \psi_{\text{AB}}=M_{\mathrm{off}}\sin\left(\omega t+\theta_{\mathrm{off}}\right). \end{array}$$

When the transformer connects to phase A and phase B, the line voltages of the transformer satisfy the condition in (13). So the flux linkages of phase BC winding and phase CA winding can be calculated in

(18) ψBC=CBC+∫ttransfert(−12UAB)dt=CBC+12ψAB,alt(t)|ttransfer−12ψAB,alt(t)=CBC+12CAB−12Moff⁡sin(ωt+θoff⁡),

(19) ψCA=CCA+∫ttransfert(−12UAB)dt=CCA+12ψAB,alt(t)|ttransfer−12ψAB,alt(t)=CCA+12CAB−12Moff⁡sin(ωt+θoff⁡).

The flux linkages of phase BC winding and phase CA winding have the same AC component, which is one-half the magnitude and 180 degrees out of phase of the AC component of phase AB winding.

So the perfect time instant to transfer phase C can be obtained by solving the equations below:

(20) CBC+12CAB−12Moff⁡sin(ωt+θoff⁡) =Moff⁡sin(ωt+θoff⁡−2π3),

(21) CCA+12CAB−12Moff⁡sin(ωt+θoff⁡) =Moff⁡sin(ωt+θoff⁡+2π3).

The equations (20) and (21) can be simplified to the equations below:

(22) $$\begin{array}{c} \cos\left(\omega t+\theta_{\mathrm{off}}\right)=\frac{-C_{\text{BC}}-\left(1/2\right)C_{\text{AB}}}{\left(\sqrt{3}/2\right)M_{\mathrm{off}}}, \end{array}$$

(23) $$\begin{array}{c} \cos\left(\omega t+\theta_{\mathrm{off}}\right)=\frac{C_{\text{CA}}+\left(1/2\right)C_{\text{AB}}}{\left(\sqrt{3}/2\right)M_{\mathrm{off}}}. \end{array}$$

Because *C*_CA_ + (1/2)*C*_AB_ equals −*C*_BC_ − (1/2)*C*_AB_, the two equations have the same solution. Therefore, there is a time instant to transfer the phase C that makes no DC component contained in the flux linkage after the transfer.

However, it is possible that the residual flux linkages satisfy the following inequality:

(24) $$\begin{array}{c} \left|C_{\text{BC}}+\frac{1}{2}C_{\text{AB}}\right|>\frac{\sqrt{3}}{2}M_{\mathrm{off}}. \end{array}$$

The equations (22) and (23) are not solvable. There is not a best time instant to transfer the phase C to eliminate the flux linkage DC component. If the phases B and C or the phases C and A are transferred first, the situation is similar.

**Figure 8:**
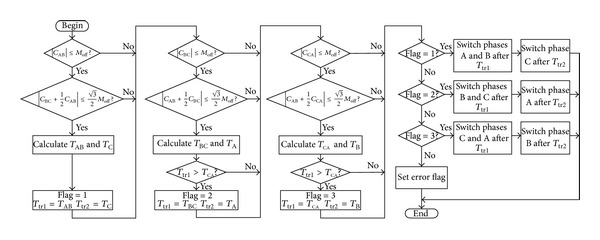
Control flowchart to transfer the alternate source.

The transfer scheme is shown in Figure 8. There are three kinds of transfer sequence: transfer phases A and B first, transfer phases B and C first, and transfer phases C and A first. After the residual flux linkages of each winding are obtained, whether the transfer sequence makes the transfer time solvable can be judged by testing the corresponding inequalities in Figure 8. In order to eliminate the DC component, the transfer sequence that makes the transfer time unsolvable will not be used. If there are two or more kinds of transfer sequence that could make the transfer time solvable, the quickest one is selected.

**Figure 9:**
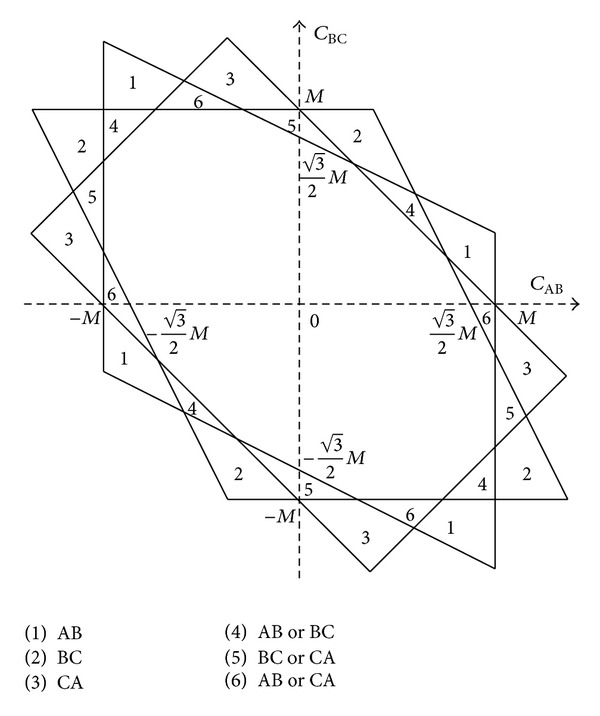
Solvable zones ((1) equations are solvable if transferring AB first, (2) equations are solvable if transferring BC first, (3) equations are solvable if transferring CA first, (4) equations are solvable if transferring AB or BC first, (5) equations are solvable if transferring BC or CA first, and (6) equations are solvable if transferring AB or CA first).

The residual flux linkages of the load transformer determine whether there are the best time instants to transfer each phase.  Figure 9 illustrates the solvable zones when each phase is transferred first. We should only select the phases to transfer first that make the equations solvable. It can be found out that when the flux linkages of each phase are unsymmetrical the equations may be unsolvable.

In article [15] the effects of the symmetrical and unsymmetrical voltage sags on the transformer were discussed. The unsymmetrical voltage sag will result in the unsymmetrical flux linkage. The unsymmetrical voltage sag is always caused by the unsymmetrical fault. For example, the two-phase short-circuit fault and the single-phase line-open fault will result in unsymmetrical residual flux linkage.

## 3. Simulations

Simulation experiments are implemented by using MATLAB to test the performance of the transfer method. The parameters are listed below.

1. Transformer: 2000 KVA, 10 kV/400 V, delta/wye connection, and nonlinear model.
2. Source: 10 kVrms line voltage, 50 Hz.
3. Load: 1600 kW, 1200 kVar.
4. Power transmission line: *L*_line_ = 5 mH, *R*_line_ = 0.2 Ω.

In the simulations, the transformer model with the two-slope saturation curve is used. The transformer model with the two-slope saturation curve is a simplified model, and this model has been adopted by many papers to test the efficiency of inrush current reduction [12].

**Figure 10:**
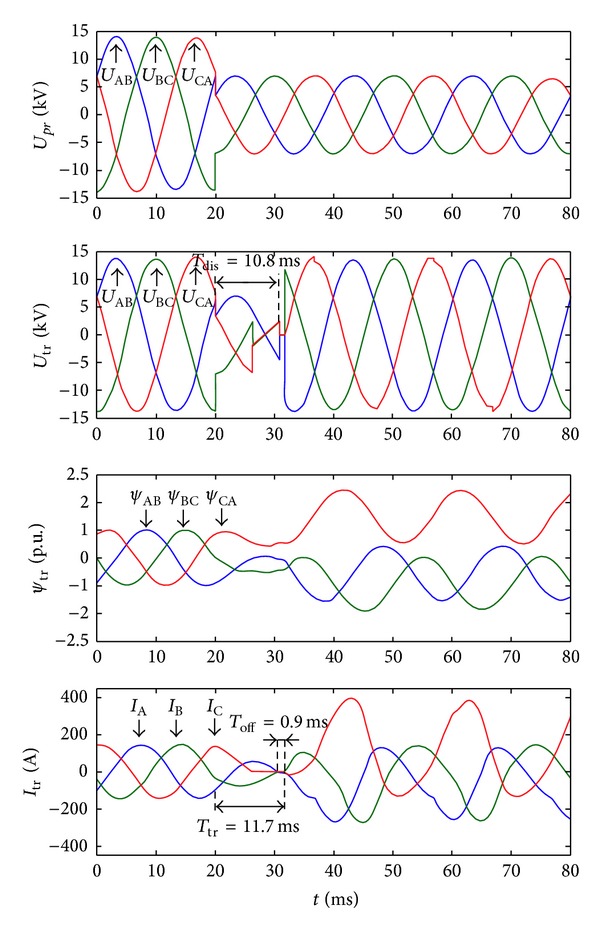
Waves of the transfer strategy without flux linkage DC component elimination (three-phase voltage sag). From the top to bottom, prefer source voltages, transformer voltages, transformer flux linkages, and load currents.

Figure 10 shows the waves of the transfer strategy without DC component elimination. The preferred source and the alternate source have the same amplitude and phase angle. The preferred source suffers a three-phase voltage sag. There is significant flux DC component which results in severe inrush current generated after the transfer.

The transfer time is totally 11.7 ms. Because the three phases of the preferred source are disconnected at different time instants, the voltage of the transformer is different with the preferred source before the preferred source is completely disconnected with the transformer. And the controller takes 0.9 ms to detect the off signal of the preferred source.

**Figure 11:**
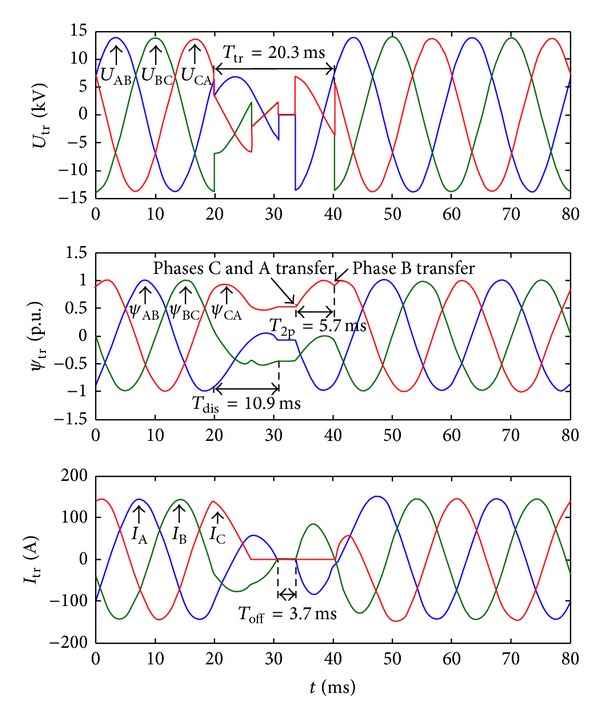
Waves of the transfer strategy with flux linkage DC component elimination (three-phase voltage sag). From the top to bottom, they are transformer voltages, transformer flux linkages, and load currents.

Figure 11 shows the waves of the transfer strategy with the flux linkage DC component elimination. It is the same fault with Figure 10. When the off signal is detected at 11.7 ms after the fault occurs, the residual flux linkage *C*_AB_ is −0.075 p.u., *C*_BC_ is −0.438 p.u., and *C*_CA_ is 0.514 p.u., and the phase angle of the prospective flux linkage is 210.6°. It can be calculated that the best time instant is to transfer the phase A and phase C after 2.8 ms and transfer the phase B after 8.5 ms. The flux linkage DC component is almost reduced to zero, and the inrush current is eliminated as a result.

It can be found out that when the phase A and phase C are connected to the transformer, the *U*_AB_ and *U*_BC_ are one-half the magnitude and 180 degrees out of phase of *U*_CA_.

The transfer time is 20.3 ms. It is 8.5 ms longer than before. And there is 5.7 ms that the transformer only connects to phase A and phase C of the alternate source.

**Figure 12:**
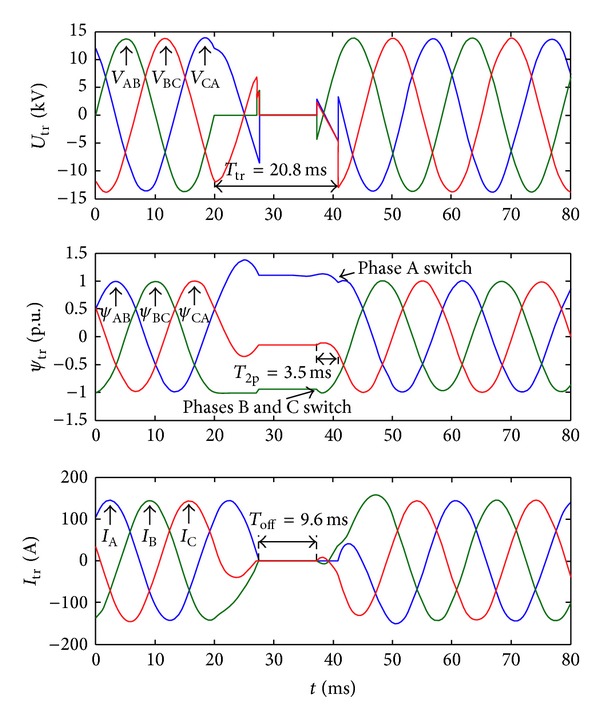
Waves of the transfer strategy with flux linkage DC component elimination. From the top to bottom, they are transformer voltages, transformer flux linkages, and load currents.

Figure 12 shows waves when there is a BC phase ground fault on the preferred source. Because the two-phase ground fault is an unsymmetrical fault, it would possibly result in the fact that there is no best time instant to transfer the third phase when some phases are transferred first.

When the off signal is detected, the *C*_AB_ is 1.081 p.u., *C*_BC_ is −0.935 p.u., and *C*_CA_ is −0.146 p.u., so that the residual flux of the three windings is in zone 2 in the fourth quadrant of Figure 7, and the best transfer time instant exists only when we transfer phases B and C first. The transfer time is 20.8 ms. There is no flux linkage DC component contained in the load transformer after the transfer.

## 4. Laboratory Experiments

**Figure 13:**
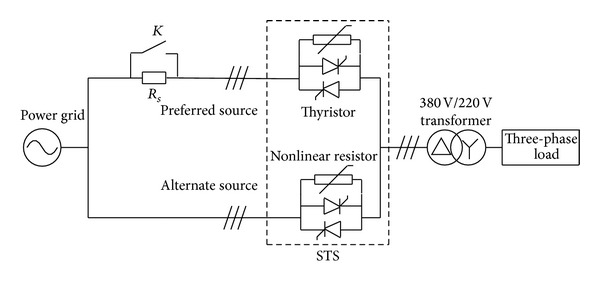
The circuit of the laboratory experiment.

**Figure 14:**
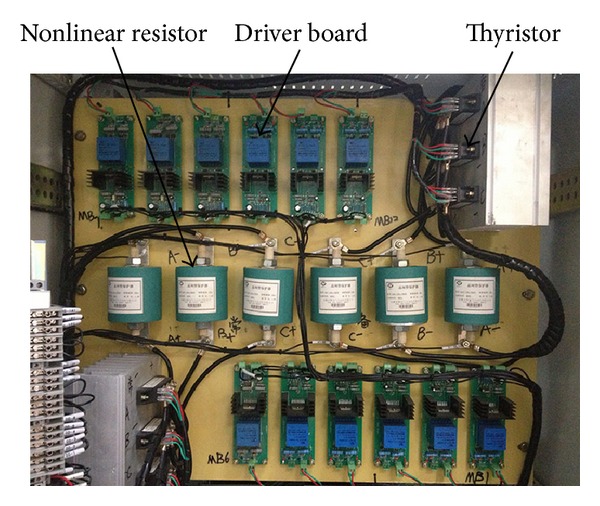
The picture of the equipment.

The experiment circuit structure is shown in Figure 13, the picture of the equipment is displayed in Figure 14, and the parameters of every component are listed below.

1. Controller: the control method is implemented in a TMS28335 DSP.
2. Source: 380 Vrms line voltage, 50 Hz.
3. Load transformer: 4 kVA, 380 V/220 V, and delta/wye connection.
4. Thyristors: MTC25A-16, 25 A, 800 V.
5. Three-phase resistance load: 47 Ω per phase, wye connection.
6. Resistance in series: *R*_*s*_ = 75 Ω.

The three-phase voltage sag on the preferred source is made by *R*_*s*_ and a parallel switch *K*.

The flux linkage is calculated by integrating of the voltage, but in the steady state, there is always slight zero drift included in the result. In order to remove the zero drift, a filter which is used to filter the DC component is added into the controller. The time constant of the filter is set to 10 seconds. The zero drift in the result can be removed by the filter in the steady state. And because the transfer time which is always tens of milliseconds is far less than the time constant of the filter, the DC offset of the flux which is produced in the transfer process can be retained before the right decision is made.

After the time instants to transfer each phase are acquired, the timers in the DSP are used to transfer each phase at the precise time instants.

**Figure 15:**
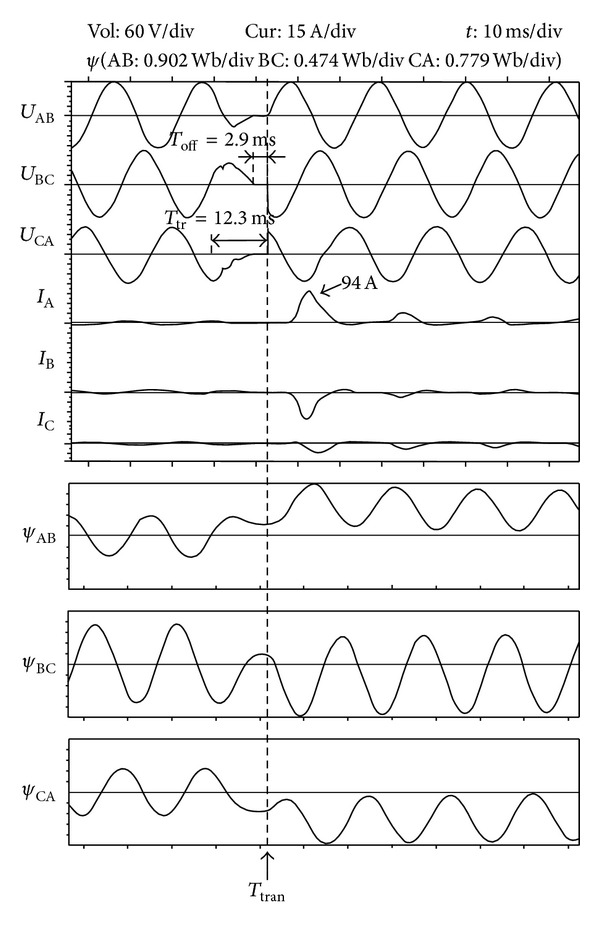
Waves of the transfer strategy without flux linkage DC component elimination (three-phase voltage sag). From the top to bottom, they are AB, BC, and CA phase transformer voltage, A, B, and C phase load current, and AB, BC, and CA phase transformer flux linkage.

Figure 15 shows the waves of the transfer strategy without the flux linkage DC component elimination. The waves of voltages and currents are recorded by the fault recorder. And the waves of the flux linkage are recorded by the DSP conntroller; the scale unit varies according to the amplitude of the flux linkage. The total transfer time is 12.3 ms. The three phases of the alternate source transfer at the same time. The flux linkage of the load transformer rises to 2.5 p.u., and the peak value of the inrush current is 94A.

**Figure 16:**
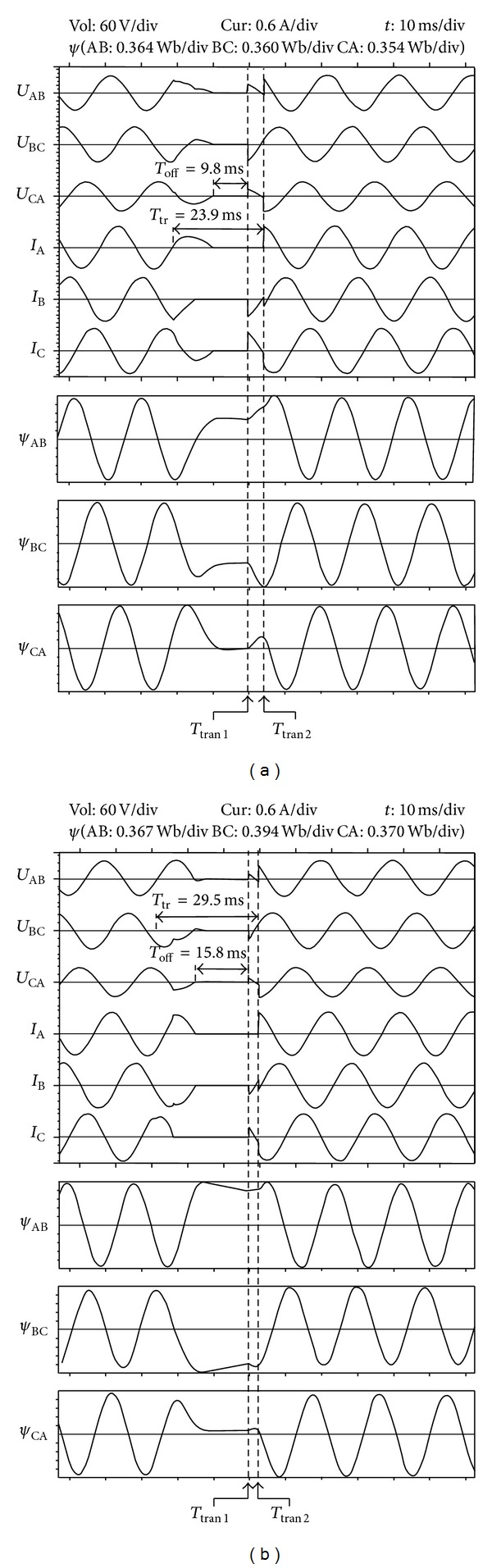
Waves of the transfer strategy with flux linkage DC component elimination. (a) Three-phase sag fault. (b) A phase line-open fault. From the top to bottom, they are AB, BC, and CA phase transformer voltage, A, B, and C phase load current, and AB, BC, and CA phase transformer flux linkage.

Figure 16(a) shows waves of the transfer strategy with the flux linkage DC component elimination when there is a three-phase fault on the preferred source. When the off signal is detected, the residual flux linkage *C*_AB_ is 0.497 p.u., *C*_BC_ is −0.470 p.u., and *C*_CA_ is −0.028 p.u., and the phase angle of the prospective flux linkage is 237.7°. It can be calculated that the best transfer scheme is to transfer phase B and phase C after 5.017 ms and transfer phase A after 9.440 ms. There is no inrush current contained in the load current. The flux linkage DC component is about 5% of the amplitude. Because of the measure error, the influence of the DC filter, and other factors, there is a little error in the result. The error after the transfer is acceptable. It can be found out that when phase B and phase C are connected to the transformer, the *U*_AB_ and *U*_CA_ are one-half the magnitude and 180 degrees out of phase of *U*_BC_.

Figure 16(b) shows waves of the transfer strategy with the flux linkage DC component elimination when there is a A phase line-open fault on the preferred source. Because the single-phase line-open fault is an unsymmetrical fault, it will result in the unsymmetrical residual flux linkage in the load transformer. When the off signal is detected, the residual flux linkage *C*_AB_ is 0.861 p.u., *C*_BC_ is −0.928 p.u., and *C*_CA_ is −0.076 p.u., and the phase angle of the prospective flux linkage is 278.8°. So the residual flux of the three windings is in zone 4 in the fourth quadrant of Figure 7. The best transfer time instant does not exist when phases A and C are transferred first. It can be calculated that the best transfer scheme is to transfer phase B and phase C after 4.960 ms and transfer phase A after 7.698 ms. The flux linkage DC component is about 7% of the amplitude. The error in the result is acceptable. And there is no inrush current contained in the load current.

The laboratory experiments results prove that the transfer method is effective to eliminate the flux linkage DC component in the load transformer.

## 5. Conclusion

This paper proposes the method to eliminate the flux linkage DC component of the load transformer for static transfer switch. The transient process of the flux linkage during the transfer interval is discussed. This method can predict the best time instants to transfer each phase according to the transient process of the flux linkage in the transformer. The results of the simulations and the laboratory experiments show the transfer method is functional.
